# Effects of intermittent negative pressure treatment on circulating vascular biomarkers in patients with intermittent claudication

**DOI:** 10.1177/1358863X211007933

**Published:** 2021-05-13

**Authors:** Henrik Hoel, Erik Mulder Pettersen, Lars Øivind Høiseth, Iacob Mathiesen, Arne Seternes, Ingebjørg Seljeflot, Jonny Hisdal

**Affiliations:** 1 Institute of Clinical Medicine, Faculty of Medicine, University of Oslo, Oslo, Norway; 2 Department of Vascular Surgery, Oslo University Hospital, Oslo, Norway; 3 Otivio AS, Oslo, Norway; 4 Department of Surgery, Sørlandet Hospital, Kristiansand, Norway; 5 Department of Circulation and Medical Imaging, Faculty of Medicine and Health Sciences, Norwegian University of Science and Technology, Trondheim, Norway; 6 Department of Anesthesiology, Oslo University Hospital, Oslo, Norway; 7 Department of Surgery, Section for Vascular Surgery, St Olavs Hospital, Trondheim, Norway; 8 Department of Cardiology, Center for Clinical Heart Research, Oslo University Hospital, Ullevål, Oslo, Norway

**Keywords:** intermittent claudication, intermittent negative pressure treatment, peripheral artery disease (PAD), vascular endothelium, vascular medicine

## Abstract

The aim of this study was to investigate the effects of lower extremity intermittent negative pressure (INP) treatment for 1 hour twice daily for 12 weeks, on circulating vascular biomarkers in patients with intermittent claudication. Patients were randomized to treatment with –40 mmHg INP (treatment group), or –10 mmHg INP (sham control group). Venous blood samples were collected at baseline and after 12 weeks, and concentrations of vascular adhesion molecule-1 (VCAM-1), intracellular adhesion molecule-1 (ICAM-1), E-selectin, P-selectin, von Willebrand factor (vWF), l-arginine, asymmetric dimethylarginine (ADMA), and symmetric dimethylarginine (SDMA) were analyzed. A larger proportion of the patients in the treatment group (25/31) had a reduction in vWF levels after 12 weeks, compared to the sham control group (17/30) (*p* = 0.043). Within the treatment group there was a significant mean (SEM) reduction in the concentration of vWF of –11% (4) (*p* = 0.019), whereas there was no significant change in the levels of vWF in the sham control group (1% (6); *p* = 0.85). There were no significant differences in the change of any of the biomarker levels between the groups after 12 weeks of treatment. In conclusion, there were no differences in the change of the circulating levels of the measured biomarkers between the treatment group and the sham control group after 12 weeks of INP treatment. However, the observed changes in vWF might indicate a beneficial effect of INP treatment on endothelial activation and endothelial injury. **Clinicaltrials.gov Identifier: NCT03640676**

## Introduction

Atherosclerosis is a multifocal disease causing build-up of atheromatous lesions in the arterial wall that may impede blood flow.^1^ In peripheral artery disease (PAD), atherosclerotic stenosis or occlusion of the arteries to the lower extremities may result in ischemic muscle pain in the legs provoked by exercise that is relieved by rest, a clinical sign known as intermittent claudication (IC).^2^

Atherosclerotic activity is associated with altered levels of circulating biochemical substances indicative of vascular inflammation, endothelial damage, endothelial dysfunction, or atheromatous plaque instability.^3–5^ In the early phase of the atherosclerotic process, the endothelium becomes activated by an atherogenic or proinflammatory stimuli, leading to upregulation and expression of adhesion molecules, recruiting monocytes and T lymphocytes to the arterial wall.^6^ Chemoattractant cytokines stimulate monocytes and T lymphocytes to enter the arterial intima,^7^ and monocytes derive into macrophages expressing receptors for internalization and oxidation of lipoproteins. The lipid loaded macrophages replicate inside the intima and secrete proinflammatory cytokines and reactive oxygen species that amplify the inflammatory response, causing progression of the atheromatous lesion.

Patients with PAD have increased risk of cardiovascular morbidity and mortality, and the aims of the treatment are twofold: first, reduction of cardiovascular risk factors; second, treatment of the leg symptoms. Standard treatment for patients diagnosed with IC is pharmacological secondary prevention with antiplatelet agents and cholesterol lowering agents, smoking cessation, and participation in supervised exercise therapy (SET) programs.^8^ A systematic review from 2014 concluded that physical activity positively affected key biomarkers in atherosclerosis,^9^ and a study from 2011 concluded that 8 weeks of SET increased walking distance, and reduced plasma levels of the specific endothelium-derived inflammatory markers E-selectin and intracellular adhesion molecule-1 in patients with PAD.^10^ However, the adherence and availability to SET programs are low,^11^ and other treatment options have been proposed. Repetitive exposure of the symptomatic leg to alternating pressure differences has been suggested to increase walking distance and improve wound healing in patients with PAD in a number of studies;^12–19^ however, as two studies did not show any additional effect on walking capacity in patients with IC, the treatment effect has been debated.^20,21^ Recently, a randomized, double blind sham-controlled trial from our research group showed that lower extremity intermittent negative pressure (INP) treatment for 1 hour twice daily for 12 weeks increased the pain-free walking distance in patients with IC.^22^ However, the physiological and biochemical mechanisms explaining the clinical improvements in patients with IC after INP treatment are not fully understood. To explore this further, we aimed to investigate the potential effect of lower extremity INP treatment for 1 hour twice daily for 12 weeks on circulating levels of vascular adhesion molecule-1 (VCAM-1), intracellular adhesion molecule-1 (ICAM-1), E-selectin, P-selectin, von Willebrand factor (vWF), l-arginine, asymmetric dimethylarginine (ADMA), and symmetric dimethylarginine (SDMA) as markers of vascular inflammation, endothelial injury, and endothelial function.

## Methods

### Participants, intermittent negative pressure (INP) treatment, randomization, and blinding

**Figure 1.**
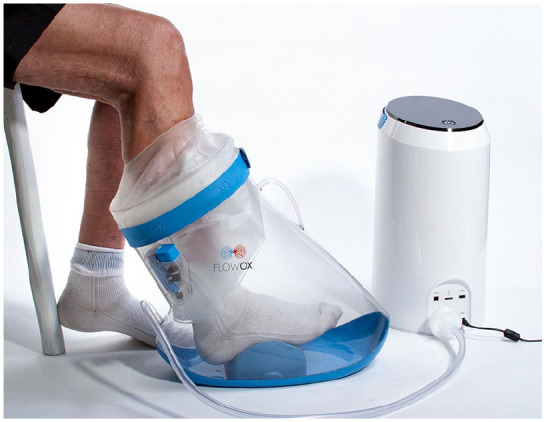
Intermittent negative pressure generated in a pressure chamber sealed around the patient’s lower leg by a pump unit that is removing air from and venting the pressure chamber. Source: Otivio AS/Bastian Fjeld.

This was an exploratory study of secondary outcome measures from a randomized controlled multicenter trial.^22^ Patients were enrolled from the outpatient clinics at three vascular surgery departments in Norway (Oslo University Hospital, Oslo; Sørlandet Hospital, Kristiansand; and St Olavs Hospital, Trondheim) between January and September 2019. Data collection was completed in December 2019. Patients with an ankle–brachial index (ABI) ⩽ 0.9, or incompressible leg arteries and radiologically diagnosed PAD, and IC were assessed for eligibility. Exclusion criteria were: endovascular or open surgical revascularization within the last 3 months, inability to perform a treadmill test, inability to independently operate the INP-treatment device, baseline maximal walking distance > 1000 m, and severe chronic obstructive pulmonary disease or severe heart disease corresponding to New York Heart Association Functional Class IV.^23^ Eligible patients were randomized to treatment with –40 mmHg INP (treatment group) or –10 mmHg INP (sham control group) in a 1:1 ratio using a computer-generated randomization list. The levels of INP used in the treatment device and in the sham device, and their impact on blood flow, has been documented in a previous study.^24^ Patients and personnel with patient contact during the study period were blinded to the group allocation, as were the laboratory technologists performing the laboratory analyses. Treatment with INP was applied in a pressure chamber sealed around the lower leg by a pump unit (FlowOx 2.0; Otivio AS, Oslo, Norway) that removed air from and vented the pressure chamber in sequences of 10 seconds negative pressure and 7 seconds atmospheric pressure (Figure 1). Pain-free and maximal walking distance were measured with a treadmill test^25^ at baseline and after 12 weeks of treatment. The patients were instructed to treat the most limiting leg at the baseline test for 1 hour in the morning and 1 hour in the evening for 12 weeks.

### Laboratory methods

Venous blood samples were collected from all patients between 08:00 and 12:00 the day before the start of the intervention period, and the day after the intervention period. Patients were instructed not to eat the same morning the samples were collected but were advised to take their regular medication with water. Serum was prepared within 1 hour by centrifugation in room temperature at 2500 × g for 15 minutes. EDTA and citrated blood were collected and stored on ice until platelet-poor plasma was obtained, and centrifugated within 30 minutes at 2800 × g for 20 minutes. All samples were frozen at –80°C. Serum was used for analysis of VCAM-1, ICAM-1, and E-selectin, citrated plasma was used for analysis of P-selectin and vWF, and EDTA-plasma was used for analysis of l-arginine, ADMA, and SDMA. Commercial ELISA kits were used for VCAM-1, ICAM-1, E-selectin, P-selectin (R&D Systems Europe, Abingdon, UK), and vWF (Asserachrom vWF Ag, Stago Diagnostica, Asnieres, France). Intra-assay coefficients of variations (CVs) were 3.3%, 2.1%, 6.5%, 3.9%, and 9.5%, respectively. l-arginine, ADMA, and SDMA were determined by high performance liquid chromatography (HPLC) and precolumn derivatization with *o*-phthaldialdehyde (OPA) (Sigma Chemicals Co., St Louis, MO, USA). CVs were 5.9%, 7.0%, and 9.6%, respectively. All samples were analyzed in batches to eliminate intra-assay variability. Routine blood samples (hemoglobin, thrombocytes, leukocytes, creatinine, total cholesterol, high-density lipoprotein cholesterol, low-density lipoprotein cholesterol, triglycerides, glycosylated hemoglobin, C-reactive protein, and albumin) were analyzed with conventional methods.

### Statistics

Data are presented as median (25th, 75th percentile) or mean (SEM) for continuous variables, and number (%) for categorical variables. Concentrations of VCAM-1, ICAM-1, E-selectin, P-selectin, vWF, l-arginine, ADMA, and SDMA at baseline were plotted against concentrations after 12 weeks of treatment. The differences in baseline and post-intervention values were dichotomized to increased (⩾ 0) or decreased (< 0), and differences in the distributions between the treatment group and the sham control group were compared using χ^2^ test. Normality was assessed with histograms, Q–Q plots, and residual plots. In the situations where data were not normally distributed, log transformations were performed. Differences in the changes of the biomarker levels between the groups were compared using analysis of covariance (ANCOVA). Differences within the groups were compared using paired sampled *t*-test. All subjects with pre- and post-data available were included in the analyses. Spearman correlation coefficients were calculated to evaluate the correlation between the change in the measured biomarkers, and the change in pain-free and maximal walking distance after 12 weeks. A *p* < 0.05 was considered statistically significant. Analyses were performed using Stata, Release 16 (StataCorp LLC, College Station, TX, USA).

As this was an exploratory study of secondary outcome measures, and clinically significant changes were difficult to estimate, a separate sample size calculation for the present study was not performed.

### Ethics

The study was approved by the Regional Committee for Medical and Health Research Ethics in Norway (ref: 2018/748) and registered on ClinicalTrials.gov (NCT03640676). Written informed consent was obtained from all patients before inclusion.

## Results

**Figure 2.**
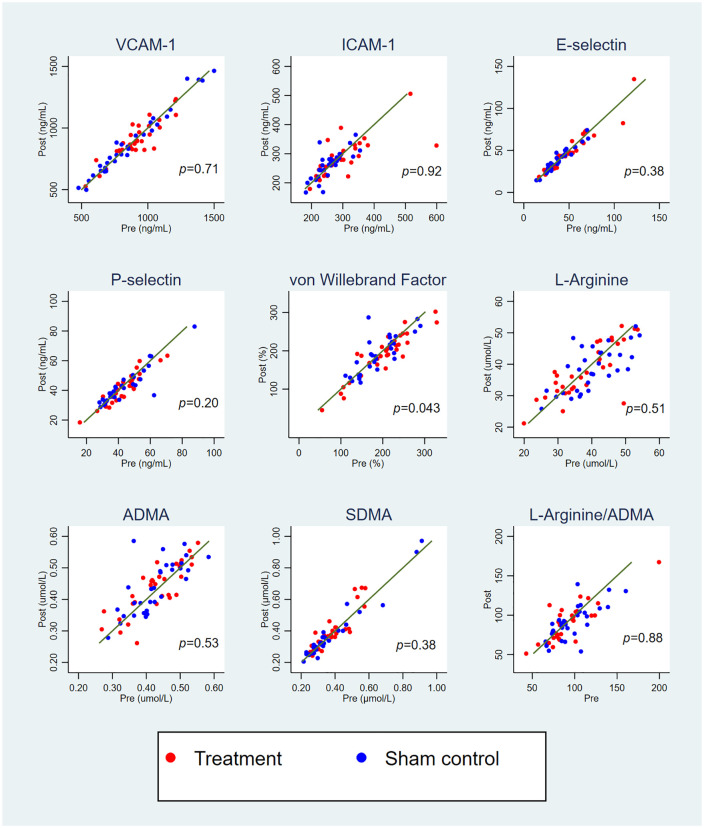
Concentrations of vascular biomarkers at baseline and after 12 weeks of intermittent negative pressure treatment. Reference lines indicating post-values = pre-values. The *p*-values refer to χ^2^ tests of proportions of patients with values increased versus decreased after 12 weeks. ADMA, asymmetric dimethylarginine; ICAM-1, intracellular adhesion molecule-1; SDMA, symmetric dimethylarginine; VCAM-1, vascular adhesion molecule-1.

In total, 85 patients were assessed for eligibility and 72 patients were randomized as part of the primary trial.^22^ Demographic variables are presented in Table 1. Sixty-three patients completed the 12-week intervention period and were available for analyses. Serum and EDTA samples were available for all patients, whereas citrated plasma samples were available for 61; thus, vWF and P-selectin analyses were lacking for two patients. For all the measured biomarkers, there was a high correlation between baseline levels and levels after 12 weeks of treatment (all pairwise Spearman’s rank correlation coefficients [*r*_s_] > 0.70). Of the patients randomized to the treatment group, 25/31 (81%) had a reduction in vWF levels after 12 weeks, compared to 17/30 (57%) in the sham control group (*p* = 0.043) (Figure 2).

**Table 1.** Baseline characteristics of patients.

Variable	Treatment (*n* = 38)	Sham control (*n* = 34)
Age, years	72 (68, 75)	73 (69, 78)
Male sex	25 (66)	26 (76)
Resting ankle–brachial index	0.50 (0.43, 0.67)	0.57 (0.46, 0.64)
Pain-free walking distance (m)	87 (45, 140)	86 (50, 151)
Maximal walking distance (m)	242 (149, 375)	236 (106, 375)
Body mass index, kg/m^2^	26.4 (24.7, 29.9)	26.7 (23.7, 29.6)
Smoking		
Current	14 (37)	11 (32)
Previous	19 (50)	18 (53)
Never	15(39)	5 (15)
Diabetes mellitus	18 (47)	6 (18)
Chronic renal failure	5 (13)	4 (12)
Hypertension	32 (84)	28 (82)
Hypercholesterolemia	22 (58)	27 (79)
Coronary artery disease	17 (45)	18 (53)
Cerebrovascular disease	8 (21)	8 (24)
Antiplatelet agent	32 (84)	27 (79)
Anticoagulant agent	6 (16)	8 (24)
Statin	32 (84)	31 (91)
Antihypertensive agent	34 (89)	31 (91)
Hemoglobin (g/dL)	14.3 (13.1, 15.0)	14.6 (13.4, 15.5)
Thrombocytes (× 10^9^/L)	256 (191, 285)	238 (183, 276)
Leucocytes (× 10^9^/L)	8.0 (6.7, 9.8)	8.1 (6.1, 8.9)
Creatinine (μmol/L)	89 (75, 115)	83 (71, 103)
eGFR (mL/min/1.73 m^2^)	67 (51, 78)	72 (61, 89)
HbA1c (mmol/mol)	44 (39, 60)	38 (36, 43)
Cholesterol (mmol/L)	3.9 (3.5, 4.4)	4.1 (3.6, 4.7)
High-density lipoprotein (mmol/L)	1.1 (1.0, 1.3)	1.3 (1.1, 1.7)
Low-density lipoprotein (mmol/L)	2.4 (1.9, 2.9)	2.3 (1.9, 2.8)
Triglycerides (mmol/L)	1.3 (1.0, 2.1)	1.3 (0.8, 1.8)
C-reactive protein (mg/L)	2 (1, 4)	2 (1, 3)
Albumin (g/L)	45 (43, 47)	44 (42, 46)

Continuous variables are presented as median (25th, 75th percentiles). Categorical variables are presented as number (%).

eGFR, estimated glomerular filtration rate; HbA1c, glycosylated hemoglobin.

**Figure 3.**
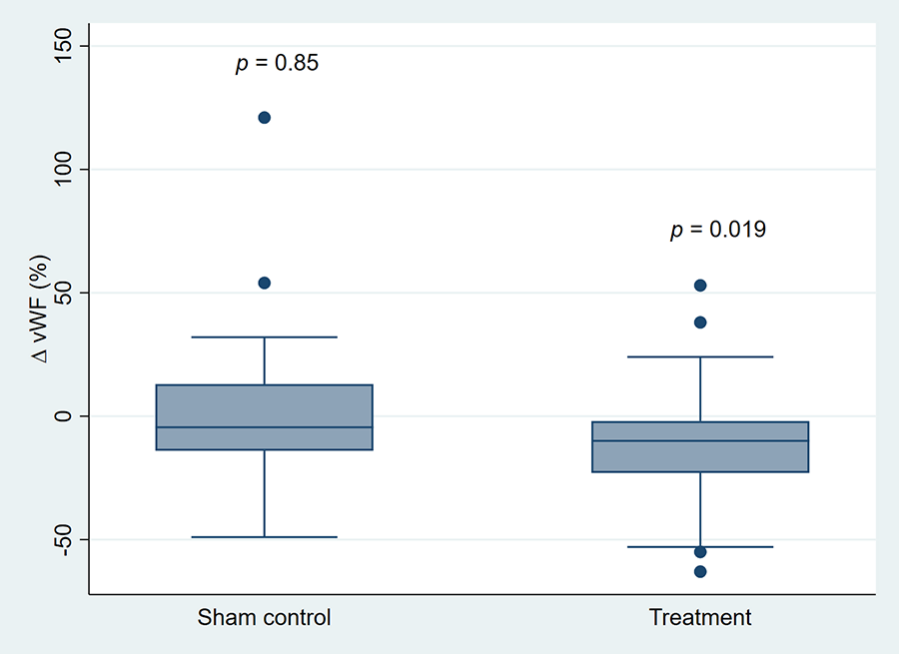
Box plot of changes in concentrations of vWF after 12 weeks of intermittent negative pressure treatment. The *p*-values are for within-group changes (paired sample *t*-test). vWF, von Willebrand factor.

There were no statistically significant differences in the change of any of the biomarker levels between the groups after 12 weeks of treatment as determined by ANCOVA (Table 2). At baseline, the mean (SEM) concentration of vWF was 200% (11) in the treatment group and 189% (9) in the sham control group. Within the treatment group there was a significant reduction in the concentration of vWF of –11% (4) (*p* = 0.019), whereas there was no significant change in the levels of vWF in the sham control group (1% (6); *p* = 0.85). The changes in vWF within the groups are illustrated in Figure 3. For all the other measured biomarkers, no significant within-group changes were shown. There was no significant correlation between the change in vWF and the change in pain-free walking distance (*r*_s_ = −0.22, *p* = 0.088), and no significant correlation between the change in vWF and the change in maximal walking distance (*r*_s_ = −0.07, *p* = 0.61) after 12 weeks.

**Table 2.** Changes in levels of circulating vascular biomarkers from baseline to 12 weeks (*n* = 63 patients).

Variable	Baseline	12 weeks	Change from baseline to 12 weeks	*p*-value within groups^a^	*p*-value between groups^b^
VCAM-1 (ng/mL)					0.53
Treatment	908 (29)	900 (28)	–8 (13)	0.58	
Sham control	879 (49)	884 (48)	4 (8)	0.61	
Log ICAM-1 (ng/mL)					0.77
Treatment	5.667 (0.043)	5.627 (0.037)	–0.039 (0.029)	0.18	
Sham control	5.553 (0.034)	5.543 (0.030)	–0.010 (0.022)	0.66	
Log E-selectin (ng/mL)					0.36
Treatment	3.739 (0.084)	3.707 (0.083)	–0.031 (0.020)	0.13	
Sham control	3.575 (0.069)	3.576 (0.070)	0.001 (0.019)	0.95	
Log P-selectin (ng/mL)					0.46
Treatment	3.721 (0.051)	3.687 (0.048)	–0.034 (0.018)	0.071	
Sham control	3.746 (0.052)	3.729 (0.047)	–0.017 (0.024)	0.47	
von Willebrand factor (%)					0.15
Treatment	200 (11)	189 (11)	–11 (4)	0.019	
Sham control	189 (9)	190 (9)	1 (6)	0.85	
l-arginine (μmol/mL)					0.56
Treatment	38 (2)	38 (1)	0 (1)	0.81	
Sham control	40 (1)	39 (1)	–2 (1)	0.13	
ADMA (μmol/mL)					0.71
Treatment	0.43 (0.01)	0.44 (0.01)	0.01 (0.01)	0.33	
Sham control	0.43 (0.01)	0.44 (0.01)	0.01 (0.01)	0.20	
Log SDMA (μmol/mL)					0.27
Treatment	–1.008 (0.046)	–0.977 (0.050)	0.031 (0.022)	0.16	
Sham control	–1.055 (0.065)	–1.054 (0.065)	0.001 (0.018)	0.95	
l-arginine/SDMA ratio					0.50
Treatment	91 (5)	89 (4)	–2 (3)	0.46	
Sham control	96 (4)	91 (4)	–6 (3)	0.06	

Data presented as mean (SEM). Log natural logarithm.

a Paired sample *t*-test.

b Analysis of covariance.

ADMA, asymmetric dimethylarginine; ICAM-1, intracellular adhesion molecule-1; SDMA, symmetric dimethylarginine; VCAM-1, vascular adhesion molecule-1.

## Discussion

The main finding of the present study was that a significantly larger proportion of the patients receiving treatment with –40 mmHg INP twice daily for 12 weeks had a reduction in vWF, compared to the patients receiving sham treatment. Further, we observed a significant reduction in the plasma concentration of vWF within the treatment group after 12 weeks; however, no differences between the groups were observed. For VCAM-1, ICAM-1, E-selectin, P-selectin, l-arginine, ADMA, and SDMA no significant changes were observed after 12 weeks of INP treatment.

In a recent paper from our research group, we concluded that INP treatment increased pain-free walking distance compared to sham treatment in patients with IC,^22^ a finding that is in line with several previous studies.^13–19^ However, to our knowledge, the present study is the first to explore the effects of INP treatment on a molecular level. vWF is a glycoprotein synthesized and stored in endothelial cells and plays important roles in primary hemostasis by mediating platelet adhesion and aggregation to sites of endothelial injury, and also mediates coagulation by stabilizing coagulation factor VIII in the circulation.^26^ Circulating levels of vWF are increased in patients with PAD^27^ and are suggested to have a prognostic value for patency after infra-inguinal bypass grafting, and for future risk of cardiovascular events.^28,29^ Although based on proportion calculations and within-group comparisons, the observed reduction in vWF after INP treatment suggests that INP treatment could reduce prothrombotic endothelial properties in patients with PAD. Exposure of the limb to INP acutely increases fluctuations in arterial and skin blood flow,^24^ leading to increased arterial shear stress followed by flow-mediated dilation.^30^ Both flow-mediated dilation and circulating levels of vWF are markers of endothelial function, and an inverse relationship between the levels of circulating vWF and the flow-mediated dilation response has been suggested.^31^ Hence, a reduction in circulating levels of vWF after INP treatment may indicate a positive effect on endothelial function and endothelial injury. There were no significant correlations between the change in the levels of vWF and the change in pain-free walking distance or maximal walking distance. A possible explanation for this finding is that the change in vWF and the change in walking distance probably represent separate effects of INP treatment.

Nitric oxide (NO) is a potent vasodilator that plays an important role in vascular homeostasis through antiatherogenic and antiproliferative effects on the arterial wall. The release of NO in response to arterial shear stress promotes flow-mediated dilation.^32^ NO is produced in the endothelial cells by the enzymatic conversion of l-arginine mediated by nitric oxide synthase (NOS). ADMA and SDMA are endogenous products of proteolysis, which inhibit NO synthesis. ADMA inhibits NOS by competing with l-arginine on the active site of NOS, while SDMA inhibits the cellular uptake of the NO precursor homoarginine. ADMA and SDMA are sensitive markers for endothelial dysfunction, and homoarginine/ADMA ratio and homoarginine/SDMA ratio are suggested to be independent predictors for long-term cardiovascular mortality and events in patients with lower extremity PAD.^33^ In the present study, we did not observe any change in l-arginine, ADMA or SDMA after 12 weeks of INP treatment. This may indicate that despite improving walking capacity in patients with IC,^22^ INP treatment does not seem to affect the NO synthesis pathway measured at a systemic level.

Atherosclerosis is a chronic inflammatory process that has predilection to discrete regions in the arterial tree where laminar blood flow is disturbed. Upregulation of adhesion molecules in response to turbulent blood flow or other proinflammatory stimuli is an important feature of the disease.^6^ Hence, circulating levels of soluble adhesion molecules such as ICAM-1, VCAM-1, E-selectin, and P-selectin may reflect the inflammatory response of the endothelium. In the present study, we did not however find any changes in the levels of these circulating adhesion molecules after 12 weeks of INP treatment. In a previous study investigating the effects of SET on endothelium-derived inflammatory markers and walking capacity in patients with IC, a significant increase in walking capacity and a significant reduction in E-selectin and ICAM-1 were observed after 8 weeks.^10^ The results from the present study indicate that INP treatment of one leg does not affect the total vascular inflammatory burden caused by atherosclerosis, in contrast to what is observed after a period with SET in patients with IC. It is therefore likely that the improvement in walking capacity observed after SET in patients with IC is related both to positive systemic effects and to local effects of exercise.

### Study limitations

There are some limitations in the present study. We did not find any significant between-group differences in the change of the levels of any of the measured biomarkers after 12 weeks of treatment. However, this exploratory study of secondary outcome measures may have been underpowered to detect such between-group differences. Hence, the change in vWF after long-term INP treatment that was observed in the present study should be verified in a larger trial. The patients were instructed to treat only their most limiting leg throughout the 12-week period. As atherosclerosis is a systemic disease, INP treatment of one leg may not have been sufficient to affect the levels of the measured biomarkers enough to show between-group effects, especially as the biomarkers are not specific to PAD.

## Conclusion

In this randomized controlled trial of patients with IC, there were no significant differences in the change in circulating levels of VCAM-1, ICAM-1, E-selectin, P-selectin, vWF, l-arginine, ADMA, and SDMA after treatment with –40 mmHg INP for 1 hour twice daily for 12 weeks, compared with sham treatment. However, a significantly larger proportion of the patients in the treatment group had a reduction in vWF compared with the sham control group, and the concentration of vWF was significantly reduced within the treatment group after 12 weeks, which might indicate a beneficial effect of INP treatment on endothelial activation and endothelial injury.
